# Population‐scale cross‐disorder atlas of the human prefrontal cortex at single‐cell resolution

**DOI:** 10.1002/alz70855_097913

**Published:** 2025-12-23

**Authors:** Jaroslav Bendl

**Affiliations:** ^1^ Icahn School of Medicine at Mount Sinai, New York, NY, USA

## Abstract

**Background:**

Neurodegenerative diseases and serious mental illnesses often exhibit overlapping molecular characteristics, underscoring the need for integrated datasets to unravel shared and distinct mechanisms. Existing studies are fragmented, employing varied cohorts and methodologies, which hinders comprehensive cross‐disorder comparisons. To address this, linking molecular, cellular, genetic, and clinical data at scale is essential for advancing our understanding of these complex conditions.

**Method:**

The PsychAD consortium has created a resource of unprecedented scale, generating single‐nucleus RNA sequencing (snRNA‐seq) and genotype data from dorsolateral prefrontal cortex samples of 1,494 human donors. This dataset spans over 6.3 million nuclei from donors with diverse diagnoses, including Alzheimer's disease, Parkinson's disease, schizophrenia, and bipolar disorder, alongside neurotypical controls. High‐throughput sequencing, rigorous quality control, and computational methods were employed to ensure reliable cell‐type‐specific profiles and facilitate cross‐disease analyses.

**Result:**

The PsychAD dataset provides high‐resolution cellular taxonomy, identifying 8 major cell classes and 67 subtypes. It offers insights into transcriptional and genetic alterations associated with neurodegenerative and psychiatric disorders and highlights shared molecular pathways. Donor metadata, such as neuropsychiatric symptom profiles and neuropathological assessments, enrich the dataset's value for targeted disease investigations. Open‐access tools enable users to explore cell‐type‐specific gene expression, identify phenotype‐associated cells, and map new datasets to the PsychAD reference framework.

**Conclusion:**

The PsychAD dataset is a transformative resource for the neuroscience community, offering integrated, high‐quality data to study neurodegenerative and psychiatric diseases. Publicly accessible through the AD Knowledge Portal, it supports collaboration and re‐use, providing a foundation for novel integrative analyses, tool development, and discovery of therapeutic targets. In this session, we will demonstrate how to leverage these data and tools for advancing research across the field.

